# Proteomic approach toward determining the molecular background of pazopanib resistance in synovial sarcoma

**DOI:** 10.18632/oncotarget.22730

**Published:** 2017-11-28

**Authors:** Zhiwei Qiao, Kumiko Shiozawa, Tadashi Kondo

**Affiliations:** ^1^ Division of Rare Cancer Research, National Cancer Center Research Institute, Chuo-ku, Tokyo 104-0045, Japan

**Keywords:** pazopanib, resistance, synovial sarcoma, tyrosine kinase

## Abstract

Pazopanib, a multitarget tyrosine kinase (TK) inhibitor, has been approved for treatment of soft tissue sarcoma. Elucidation of the molecular background of pazopanib resistance should lead to improved clinical outcomes in sarcomas; accordingly, we investigated this in synovial sarcoma using a proteomic approach. Pazopanib sensitivity was examined in four synovial sarcoma cell lines: SYO-1, HS-SYII, 1273/99, and YaFuSS. The 1273/99 cell line showed significantly higher IC_50_ values than the others for pazopanib. Expression levels of 90 TKs in the cell lines were examined by western blotting. Among these, the levels of PDGFRB, DDR1, AXL, MET, and PYK2 were higher, and those of FGFR1 and VEGFR3 were lower in the 1273/99 cell line than the other cell lines. Gene silencing analysis of the TKs upregulated in 1273/99 cells showed differing effects on cell growth: *PDGFRB*, *MET,* and *PYK2* knockdown induced cell growth inhibition, whereas *DDR1* and *AXL* knockdown did not influence cell growth. Using the PamChip peptide microarray, we found that 18 peptide substrates were highly phosphorylated in the 1273/99 cell line compared with other cell lines. Using the PhosphoNet database, we found that kinases FGFR3, RET, VEGFR1, EPHA2, EPHA4, TRKA, and SRC phosphorylated these 18 peptide substrates. Moreover, the results for overexpressed and aberrantly activated TKs in pazopanib-resistant cells showed no overlap. Taken together, our study indicates that identification of comprehensive TK profiles represents an essential approach to determining the molecular background of pazopanib resistance in synovial sarcoma.

## INTRODUCTION

Pazopanib is a molecular targeted drug approved by the US Food and Drug Administration for soft-tissue sarcoma [1]. It is a multitarget tyrosine kinase (TK) inhibitor (TKI) with activity against vascular endothelial growth factor receptor (VEGFR)-1, VEGFR-2, and VEGFR-3; platelet-derived growth factor receptor (PDGFR)-A and PDGFRB; fibroblast growth factor receptors; and KIT [2-4]. Based on the results of a clinical trial, namely, pazopanib for metastatic soft-tissue sarcoma (PALETTE), pazopanib is currently recommended as the gold standard treatment after failure of standard chemotherapy for patients with metastatic non-adipocytic soft tissue sarcoma [5]. However, pazopanib resistance represents a major hurdle to improving the overall response and survival of patients with soft-tissue sarcoma. The lack of understanding of the molecular background of resistance represents a major challenge in the treatment of soft-tissue sarcoma using pazopanib.

Proteomic analysis is an essential approach for the identification of proteins associated with drug resistance [6, 7]. In particular, the comparative proteomic approach has been successfully applied to the discovery of drug resistance mechanisms [8, 9]. Among proteomic techniques, antibody-based proteomics plays an important role in the identification and validation of new cancer biomarkers associated with drug resistance [10-12]. In addition, kinase activity profiling enables the detection of kinase activity in cell lysates via analysis of the level of substrate phosphorylation; this provides a comprehensive picture of drug resistance–related kinases [13, 14]. Recently, a high-throughput kinase activity screening tool, the PamChip peptide microarray system, which contains a porous microarray with 144 kinase substrates, has been used to identify the TK activity profile in many types of cancers [15-18]. A critical step for overcoming resistance against TKIs is to obtain comprehensive understanding of their molecular background; the use of TKI-centric strategies would accelerate this.

To elucidate the molecular background of pazopanib resistance in sarcoma, we examined the protein expression levels of 90 TKs by western blotting. Further, we performed TK activity profiling in synovial sarcoma cell lines using the PamChip peptide microarray. Via this approach, we identified TKs whose expression or activity was considerably different.

## RESULTS

### Sensitivity of the four cell lines to pazopanib treatment

**Figure 1:**
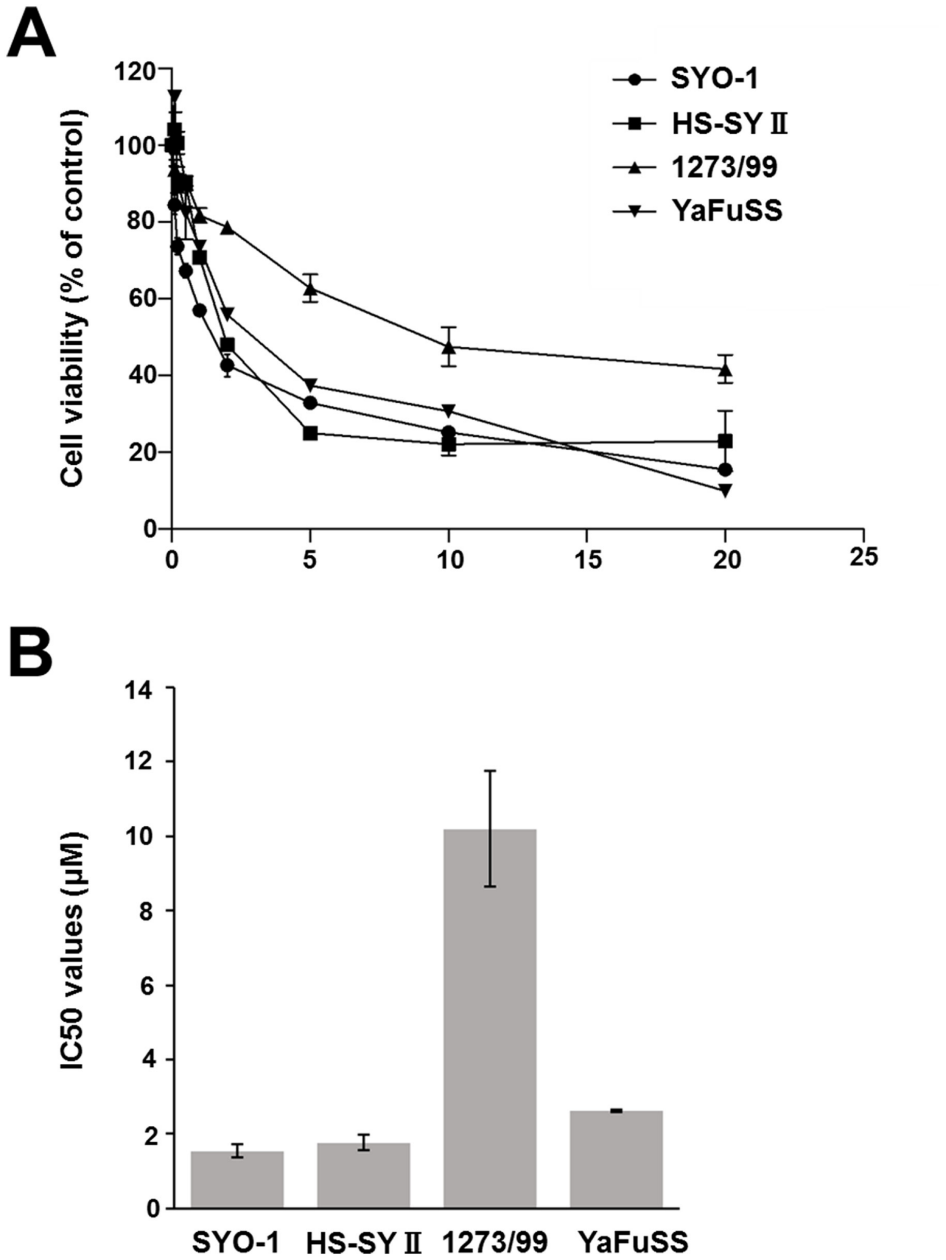
Effect of pazopanib on proliferation of synovial sarcoma cells **(A)** Four synovial sarcoma cell lines were incubated in the presence of pazopanib (0, 0.2, 0.5, 1, 2, 5, 10, or 20 μM) for 72 h. The relative number of remaining cells was evaluated by CCK-8 assay. Values represent mean ± SD; ^*^p < 0.05. **(B)** Bar graph shows the IC_50_ values of pazopanib in the four synovial sarcoma cell lines.

To verify the sensitivity of synovial sarcoma cells to pazopanib, the four synovial sarcoma cell lines, namely SYO-1, HS-SYII, 1273/99, and YaFuSS, were treated with various concentrations of pazopanib for 72 h, and cell viability was determined. The percentage of surviving cells decreased in a dose-dependent manner in all the cell lines (Figure 1A). The 1273/99 cell line was more resistant to pazopanib than the other three cell lines. The 72-h half maximal inhibitory concentrations (IC_50_) of pazopanib for the SYO-1, HS-SYII, 1273/99, and YaFuSS cells were 1.41 ± 0.19, 1.92 ± 0.22, 10.3 ± 1.45, and 2.59 ± 0.05 μM, respectively (Figure 1B).

### TKs associated with pazopanib resistance in synovial sarcoma cell lines

**Figure 2:**
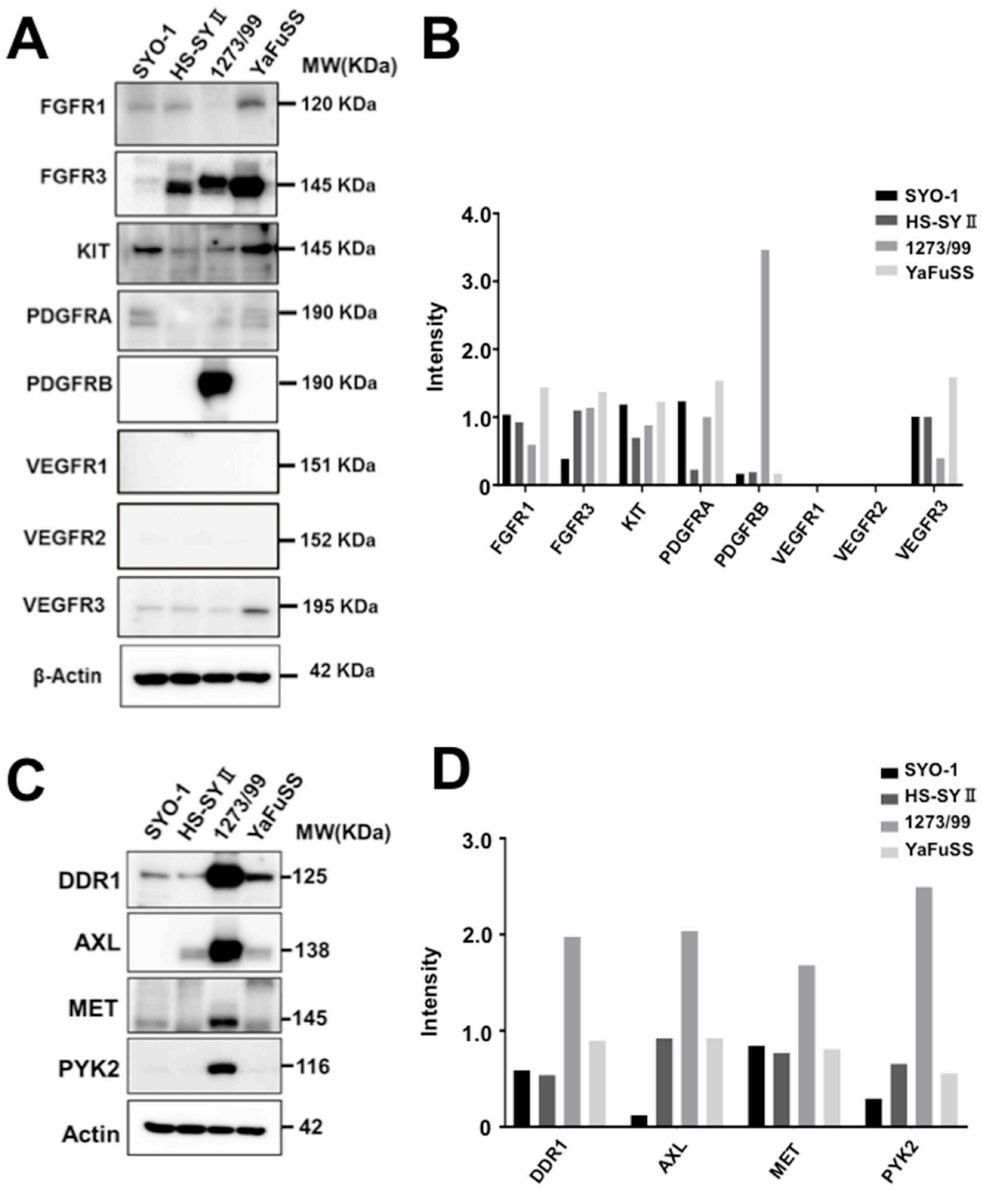
Protein expression levels of targets of pazopanib and other TKs that were differentially expressed in pazopanib-resistant cells All TK antibodies were used to detect the protein expression levels of 90 TKs in four synovial sarcoma cell lines. The expression levels of targets of pazopanib are shown as **(A)**, and the relative intensity is shown as a bar graph **(B)**. The other TKs that were differentially expressed (fold change > 1.5) in the pazopanib-resistant cells than other cells are shown as **(C)**, and the relative intensity is shown as a bar graph **(D)**.

To identify TKs whose expression levels were related to pazopanib resistance, the expression levels of 90 TKs in four synovial sarcoma cell lines were examined by western blotting. The expression levels of 44 of 90 TKs could be identified. The results have been shown in a heatmap format (Supplementary Figure 1). The protein expression levels of targets of pazopanib (FGFR-1, FGFR-3, KIT, PDGFRA, PDGFRB, VEGFR-1, VEGFR-2, and VEGFR-3) were compared; the results are shown as Figure 2A. We found that the FGFR-1 and VEGFR-3 expression levels were lower and PDGFRB expression level was higher in the 1273/99 cell line than in the other cell lines (fold change > 2; Figure 2B). Furthermore, we compared the expression levels of TKs that were not targets of pazopanib in 1273/99 cells with those in the other cell lines. We found that discoidin domain receptor (DDR)1, AXL, MET, and proline-rich tyrosine kinase (PYK)2 were expressed at higher levels in the 1273/99 cell line than in the other cell lines (fold change > 2; Figure 2C, 2D). To verify the TKs associated with pazopanib resistance, we also performed microarray analysis to validate the variations in gene expression levels of these TKs. The gene expression levels of 44 of 90 TKs are shown in supplementary Figure 2A. We compared the mRNA and protein expression data, and calculated the correlation coefficient between them. We found that all differentially expressed tyrosine kinases, excepted VEGFR3, showed a high correlation coefficient between mRNA and protein expression data (Supplementary Figure 2B, 2C).

### Functional properties of overexpressed TKs in pazopanib-resistant cells

**Figure 3:**
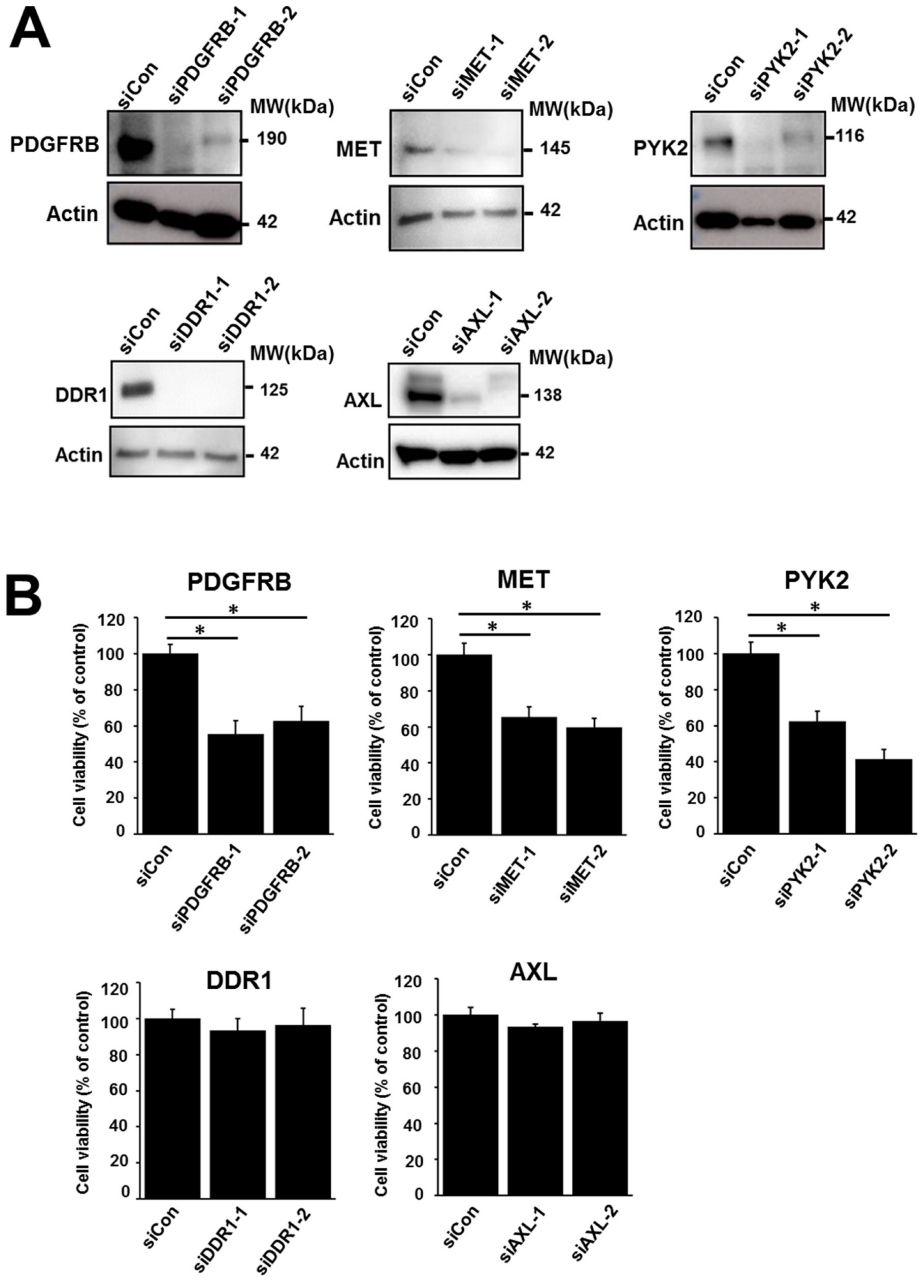
Effect of siRNA-mediated gene silencing of upregulated TKs on proliferation of the 1273/99 cell line Validation of knockdown effect of siRNA by western blotting **(A)**. Effect of siRNA-mediated gene silencing of *PDGFRB*, *MET*, *PYK2*, *DDR1*, and *AXL* on cell proliferation. Cell viability was measured after 72 h **(B)**.

To examine the functional properties of the TKs overexpressed in the 1273/99 cells, we performed gene silencing assays and measured cell proliferation. Using western blotting, we confirmed that the expression levels decreased after transfection with small interfering RNAs against the overexpressed TKs (PDGFRB, MET, PYK2, DDR1, and AXL; Figure 3A). Then, we examined the viability of 1273/99 cells after siRNA treatment for 72 h. We found that proliferation of 1273/99 cells was inhibited by siPDGFRB, siMET, and siPYK2. siAXL and siDDR1 did not affect the proliferation of 1273/99 cells (Figure 3B). In addition, we examined the effect of these five siRNAs on proliferation in the SYO-1, HS-SY2, and YaFuSS cell lines, and found that they were not affected (Supplementary Figure 3). Moreover, we examined the synergistic effect of knockdown of the overexpressed TKs and pazopanib treatment in 1273/99 cells. The 1273/99 cells were treated with siRNA and various concentrations (0.2–20 μM) of pazopanib, and cell proliferation was determined. No synergistic effects were identified between knockdown of any of the overexpressed TKs and pazopanib treatment (Supplementary Figure 4).

### TK activity in the cell lines

**Figure 4:**
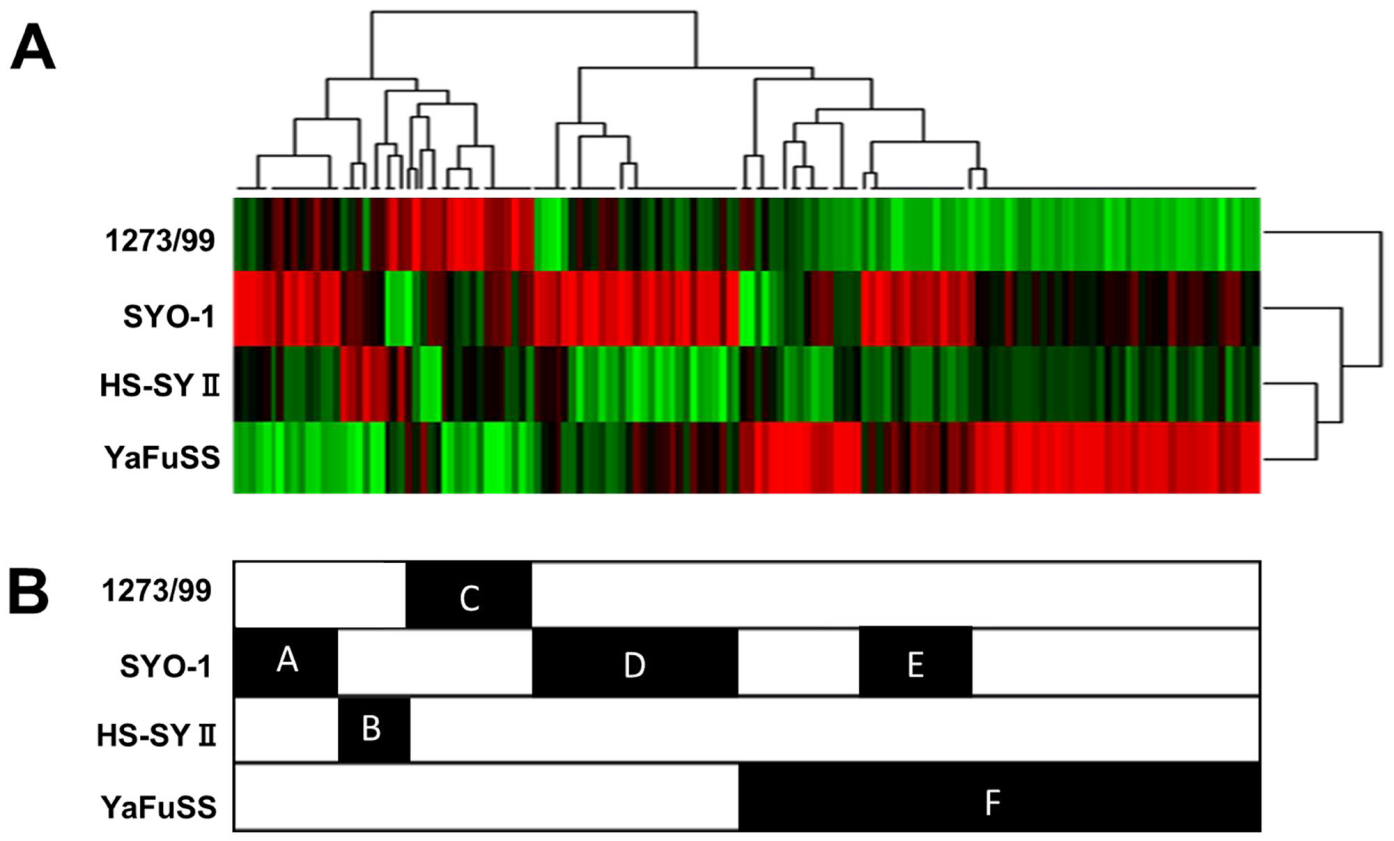
Basal tyrosine kinase activity profiling of four synovial sarcoma cell lines Protein tyrosine kinase activity profile obtained using four synovial sarcoma cell lysates, as tested on PamChip microarrays. The color-coded signature is shown as a heatmap in which high level of kinase activity is reflected by red and low level by green.

In order to perform TK activity profiling in the four synovial sarcoma cell lines, the TK activity in lysates from these lines was analyzed using kinase PamChip peptide microarrays. TK activity profiling data are shown as an unsupervised hierarchically clustered heat map of log signal intensity for each phosphosubstrate (Figure 4A). The result obtained using the PamChip peptide microarray were clustered according to peptides and cell lines along the x- and y-axes, respectively. Six major clusters (clusters A, B, C, D, E, and F) of peptides were identified (Figure 4B). Using the 18 peptides in cluster C that were highly phosphorylated in 1273/99 cells, we analyzed the kinases that phosphorylate these peptides, using the PhosphoNet database. We found that these kinases were FGFR3, rearranged during transfection (RET), VEGFR1, EPHA2, EPHA4, TRKA, and SRC (Supplementary Table 3). On comparing these kinases with overexpressed TKs identified by western blotting in 1273/99 cells, we found no overlap between them.

## DISCUSSION

In this study, we focused on 90 TKs and examined their expression levels in four synovial sarcoma cell lines with varying sensitivities to pazopanib. 44 tyrosine kinases were identified by western blot, and the tyrosine kinases coverage closed to 50% in this proteomic study. We additionally performed kinase activity profiling to identify TKs with high levels of activity associated with pazopanib resistance. The present study led to the identification of TKs whose expression or activity was considerably different. Our findings showed that analysis of TK profiles is an important approach for elucidating the molecular background of pazopanib resistance in sarcoma. Further, these findings aid in exploring the mechanism underlying TKI resistance in this form of cancer.

To examine the function of overexpressed TKs in pazopanib-resistant cells, we performed a gene silencing assay and evaluated the effects on cell proliferation. We found that not all overexpressed TKs were involved in pazopanib resistance in synovial sarcoma cells. Furthermore, we used a peptide microarray system (PamGene) to examine the kinase activity among the four cell lines, and found that the kinases in pazopanib-resistant cells were not consistent with the overexpressed TKs identified by western blotting. We considered that the kinase activity of some overexpressed TKs was not regulated by protein expression in synovial sarcoma cells. Previous studies have shown that kinase activity is not regulated only by protein expression: Lutz et al. found that Src kinases participate in growth regulation of pancreatic cancer cells, and that the kinase activity level of Src is not regulated by protein expression levels [25]. In light of the above-mentioned findings, we suggested that identification of comprehensive TK profiles is a crucial approach for investigating the molecular mechanism underlying pazopanib resistance in synovial sarcoma.

We examined the kinase activity profiling of synovial sarcoma cell lines using a commercially available array (PamChip) consisting of 144 protein TK substrates. Several studies have discussed the potential of this array for target identification in clinical samples [26], and others have proposed the application of this system to predict response to TKIs [27]. In the current study, using this system, we identified FGFR3, RET, VEGFR1, EPHA2, EPHA4, TRKA, and SRC as kinases associated with pazopanib resistance. Some of the TKs identified in this study have been previously reported to be involved in drug resistance in cancer: Girotti et al. reported that SRC is a potential target in drug-resistant BRAF mutant melanoma [28]. Bianco reported that WEGFR1 contributes to EGFR inhibitor in human cells [29]. Zheng et al. showed that RET is associated with drug resistance in colorectal cancer [30]. Zhang et al. reported that increase in EPHA2 expression mediates resistance to trastuzumab therapy in breast cancer [31]. Further studies are required to address the role of TKs associated with pazopanib resistance in synovial sarcoma.

Previous studies have reported that kinome profiling is a highly useful approach for elucidating the molecular mechanisms underlying drug resistance in cancer. Cox et al. reported that alterations in the kinome affected drug resistance in leukemia [32]. Kurimchak et al. showed that resistance to human bromodomain and extraterminal domain (BET) bromodomain inhibitors is mediated by kinome reprogramming in ovarian cancer [33]. Cooper et al. reported that dysregulated kinases and kinome dynamics in cancer cells are important targets for overcoming drug-resistant leukemia [34]. In the current study, using western blotting and the PamChip peptide microarray, we identified TKs that were associated with pazopanib resistance in synovial sarcoma. The use of a combination of antibody-based and activity-based proteomics for tyrosine kinases should deepen our understanding of drug resistance in sarcoma.

The current study has some limitations. First, an important consideration in the selection of cell lines for drug response is the ethnic background of the individuals from whom the cell lines have been derived. Numerous potential issues result from population heterogeneity. In this study, SYO-1, HS-SY II, and YaFuSS cell lines were derived from Japanese patients, while the 1273/99 cell line was derived from a European patient. We did not examine the effect of cell origin as sufficient synovial sarcoma cell lines are not available. Second, the results for kinase activity were not validated by other proteomic approaches, such as mass spectrometry-based phosphoproteomics. Further research is required to validate these results.

In conclusion, our study demonstrated that identification of comprehensive TK profiles is an essential approach for determining the molecular background of pazopanib resistance in synovial sarcoma.

## MATERIALS AND METHODS

### Cells and culture

Four synovial sarcoma cell lines were used in this study: SYO-1 was a gift from Akira Kawai (National Cancer Center, Tokyo, Japan) [19], HS-SY-II from Hiroshi Sonobe (Kochi Medical School, Kochi, Japan) [20], 1273/99 from Olle Larsson (Karolinska Institute, Stockholm, Sweden) [21, 22], and YaFuSS from Tatsuya Ishibe (University of Tokyo, Tokyo, Japan) [23]. Low-glucose Dulbecco’s modified Eagle’s medium (DMEM) supplemented with 10% fetal bovine serum (FBS) was used to culture cells. Cells were cultured at 37°C in a humidified atmosphere with 5% CO_2_. Cells from exponentially growing cultures were used in all experiments.

### Cell viability analysis

The tumor cells were plated on 96-well plates (10,000 cells/well) and incubated in the presence of pazopanib (0, 0.2, 0.5, 1, 2, 5, 10, or 20 μM) for 72 h. Then, cell growth was measured using the Cell Counting Kit-8 (CCK-8; Dojindo Laboratories, Kumamoto, Japan) according to the manufacturer’s recommendations.

### Western blotting

Each protein sample (5 μg) was subjected to separation using sodium dodecyl sulfate (SDS)–polyacrylamide gel electrophoresis (PAGE) on a 12.5% polyacrylamide gradient gel; this was followed by blotting onto a nitrocellulose membrane. Immunoblot analysis was performed using the antibodies listed in Supplementary Table 1 and horseradish peroxidase–conjugated secondary IgG antibodies (1 : 2,000; Sigma, St Louis, MO, USA). Antibody–antigen complexes were visualized with an ECL Prime System (GE Healthcare, Milwaukee, WI, USA) using the Amersham Imager 600 (GE Healthcare). The protein band intensity was quantified, and the relative intensity of the proteins examined was calculated on the basis of the actin band intensity on the same membrane. Band intensity was measured using ImageJ (US National Institutes of Health, Bethesda, MD, USA).

### Microarray analysis

Total RNA was extracted from synovial sarcoma cell lines using the RNeasy kit (Qiagen, Venlo, the Netherlands). In brief, mRNA expression profiles of the samples were obtained by hybridizing the RNA to the SurePrint G3 Human GE DNA microarray (8×60K, Ver3.0, Agilent Technologies, Santa Clara, CA), following the manufacturer’s instructions. Hybridized microarrays were scanned with a microarray scanner (Agilent G2565BA) with default protocols and settings. The microarray data were normalized and standardized using the Bioconductor agilp limma package (http://bioconductor.org/packages/agilplimma/). Differences between two sample groups were established using an unpaired t-test. A p-value < 0.05 was considered to represent statistical significance.

### Gene silencing assay

siRNAs were purchased from Sigma, and non-specific control siRNA duplexes (AllStar Negative Control siRNA) were purchased from Life Technologies. The target sequences have been provided in Supplementary Table 2. A total of 1 × 10^4^ synovial sarcoma cells were seeded into each well of the 96-well plate. On the following day, the cell monolayer was washed with prewarmed sterile phosphate-buffered saline. The cells were transfected with the appropriate siRNA by using RNAi max transfection reagents (Thermo Fisher, Waltham, MA, USA) in accordance with the manufacturer’s protocol. Twenty-four hours later, the culture medium was replaced with DMEM-low glucose (Sigma). The cells were harvested for western blotting or treated with pazopanib for 72 h after transfection.

### TK activity profiling using PamChip peptide microarrays

TK activity profiles were determined using the PamChip TK peptide microarray system (PamGene International B.V’s-Hertogenbosch, The Netherlands), as described previously [24]. Briefly, total synovial sarcoma cells were lysed in M-PER Mammalian Extraction Buffer (Pierce, Rockford, IL, USA). Cleared cell lysate (5 μg) was mixed with 4 μl of 10 × protein TK reaction buffer (PK), 0.4 μl of 1 M dithiothreitol, 0.4 μl of 100 × bovine serum albumin, 1 μl of 4 mM ATP, and 0.3 μl of 1 mg·ml^−1^ monoclonal anti-phosphotyrosine FITC conjugate (clone PY20); the total volume was adjusted to 40 μl with distilled H_2_O. All chemicals were provided by PamGene International BV. Each array was blocked with 0.2% bovine serum albumin and washed with PK solution. A kinase reaction was then performed at 30 °C. The reaction mix was pulsed back and forth through the porous material of the PamChip for 60 cycles. An image was obtained with a built-in CCD camera every fifth cycle. The study was performed in triplicate. Kinases that phosphorylate substrate peptides were predicted by referring to Phospho.elm (http://phospho.elm.eu.org) and Uniprot (http://www.uniprot.org) databases.

## SUPPLEMENTARY MATERIALS FIGURES AND TABLES








