# Supplementary material for: Proteomic approach toward determining the molecular background of pazopanib resistance in synovial sarcoma
Source: Oncotarget. 2017 Nov 28;8(65):109587–95. doi: 10.18632/oncotarget.22730 (PMC5752544; doi:10.18632/oncotarget.22730)
Supplement: Supplementary file 1 [file oncotarget-08-109587-s001.pdf]

Proteomic approach toward determining the molecular background of pazopanib resistance in synovial sarcoma

SUPPLEMENTARY MATERIALS

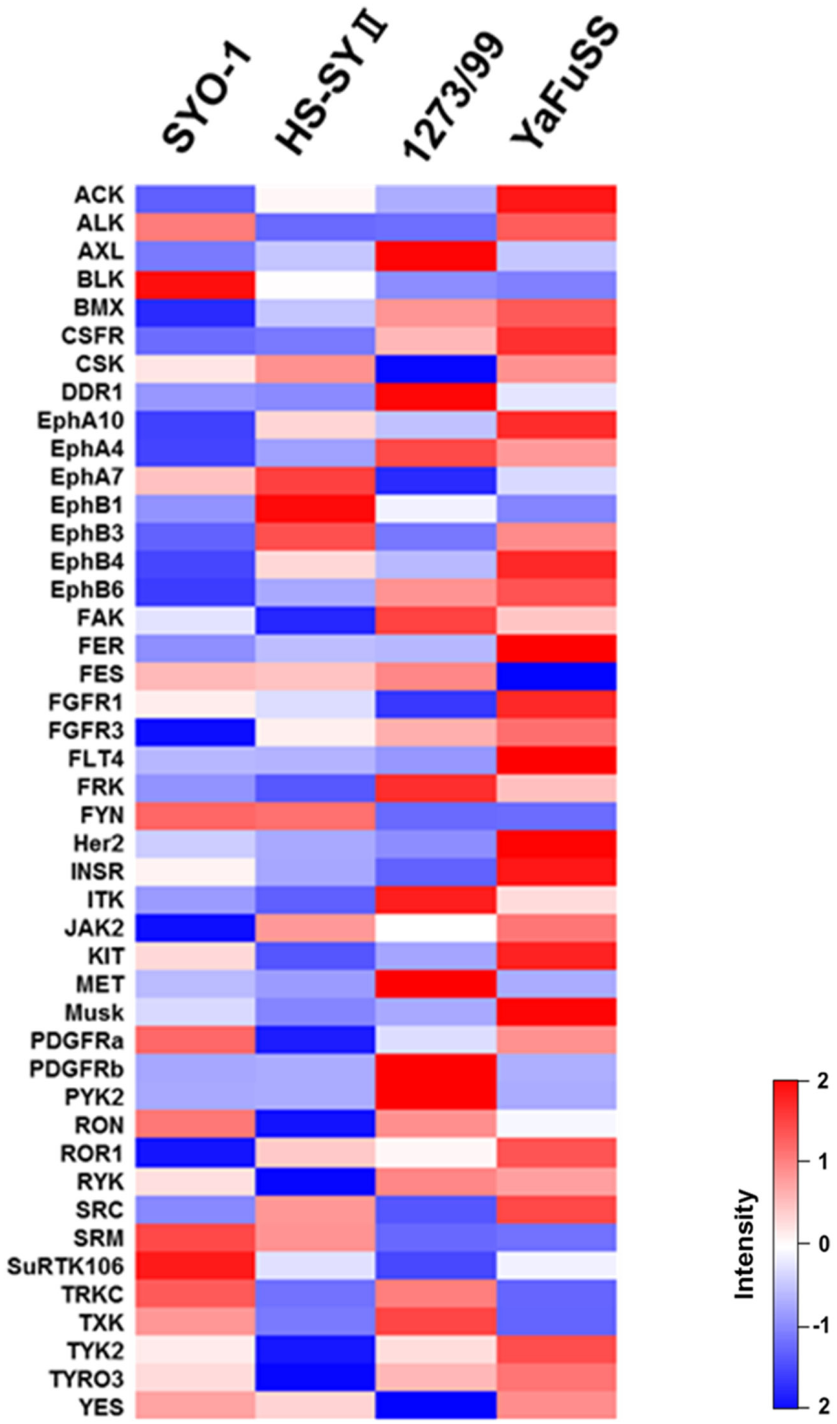

Supplementary Figure 1: Protein expression levels of 44 TKs identified by western blotting; the expression levels were normalized and are shown as a heatmap.

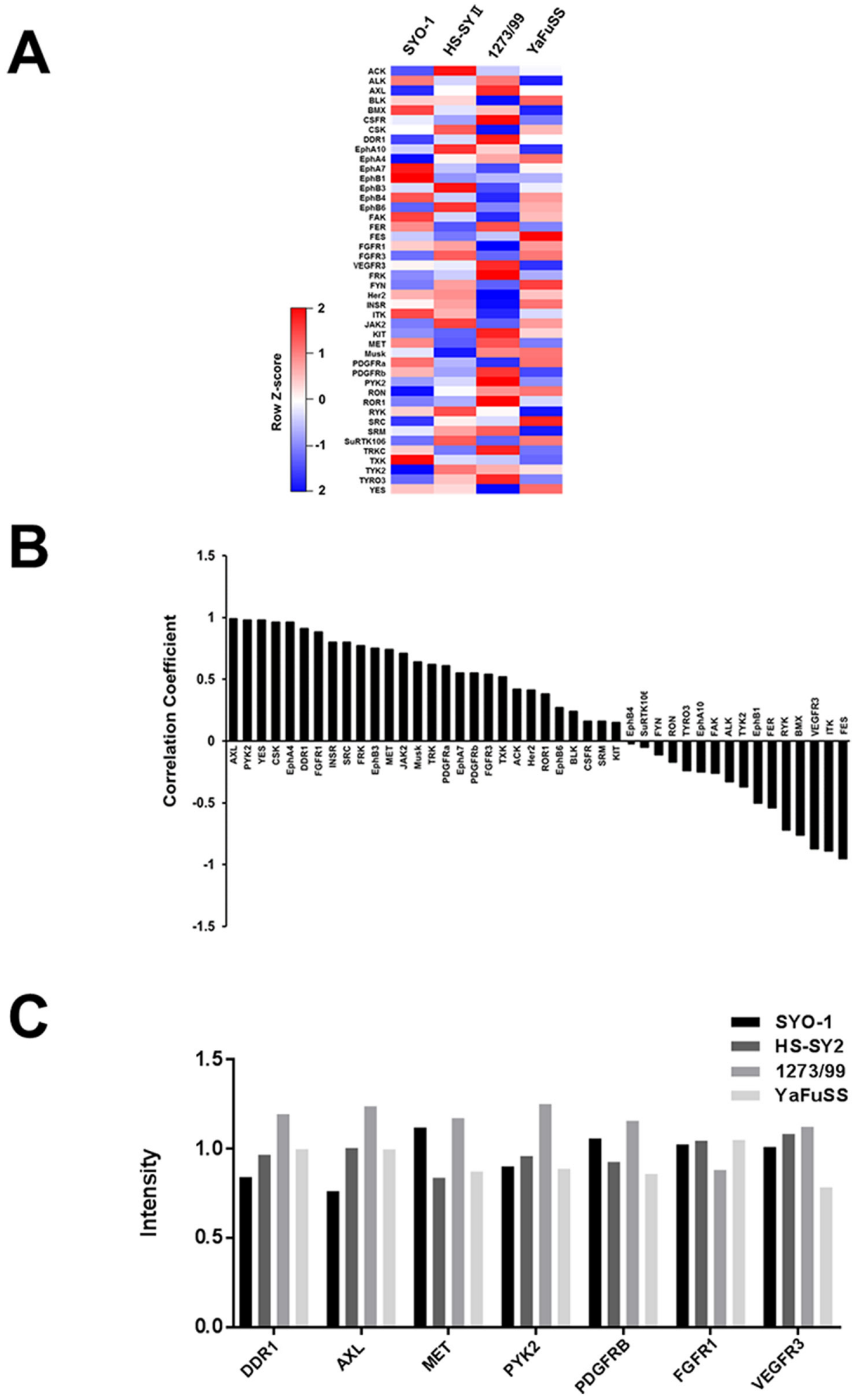

Supplementary Figure 2: Gene expression levels of 44 TKs identified by microarray; the expression levels were normalized and are shown as a heatmap. (A). Correlation coefficients between protein and mRNA expression levels are shown in **(B)**, and the mRNA expression levels of highly expressed tyrosine kinases in 1273/99 cells are shown in **(C)**.

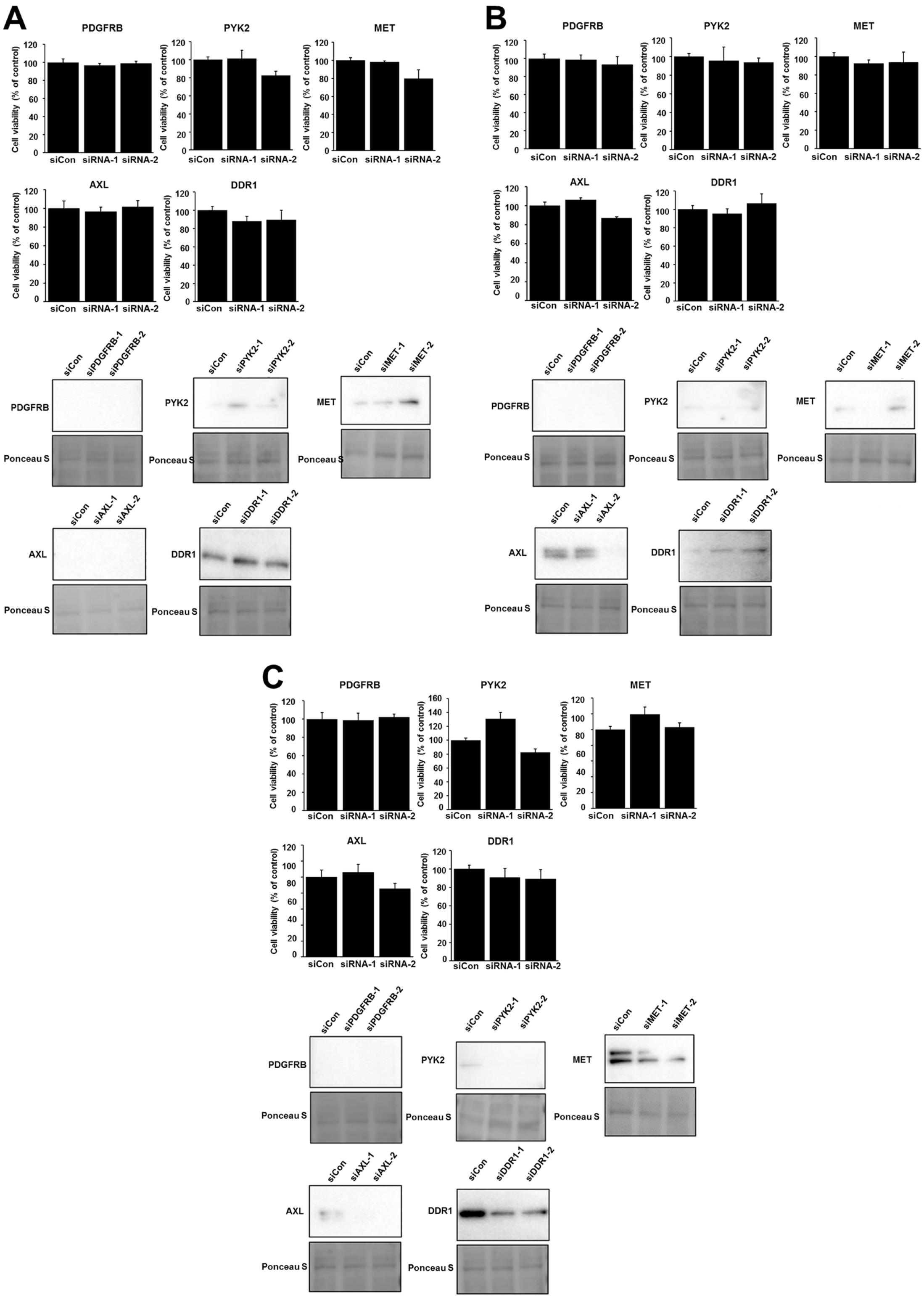

Supplementary Figure 3: Effect of siRNA-mediated gene silencing of upregulated TKs on cell proliferation in three synovial sarcoma cell lines: SYO-1 **(A),** HS-SYΠ **(B),** and YaFuSS **(C).**

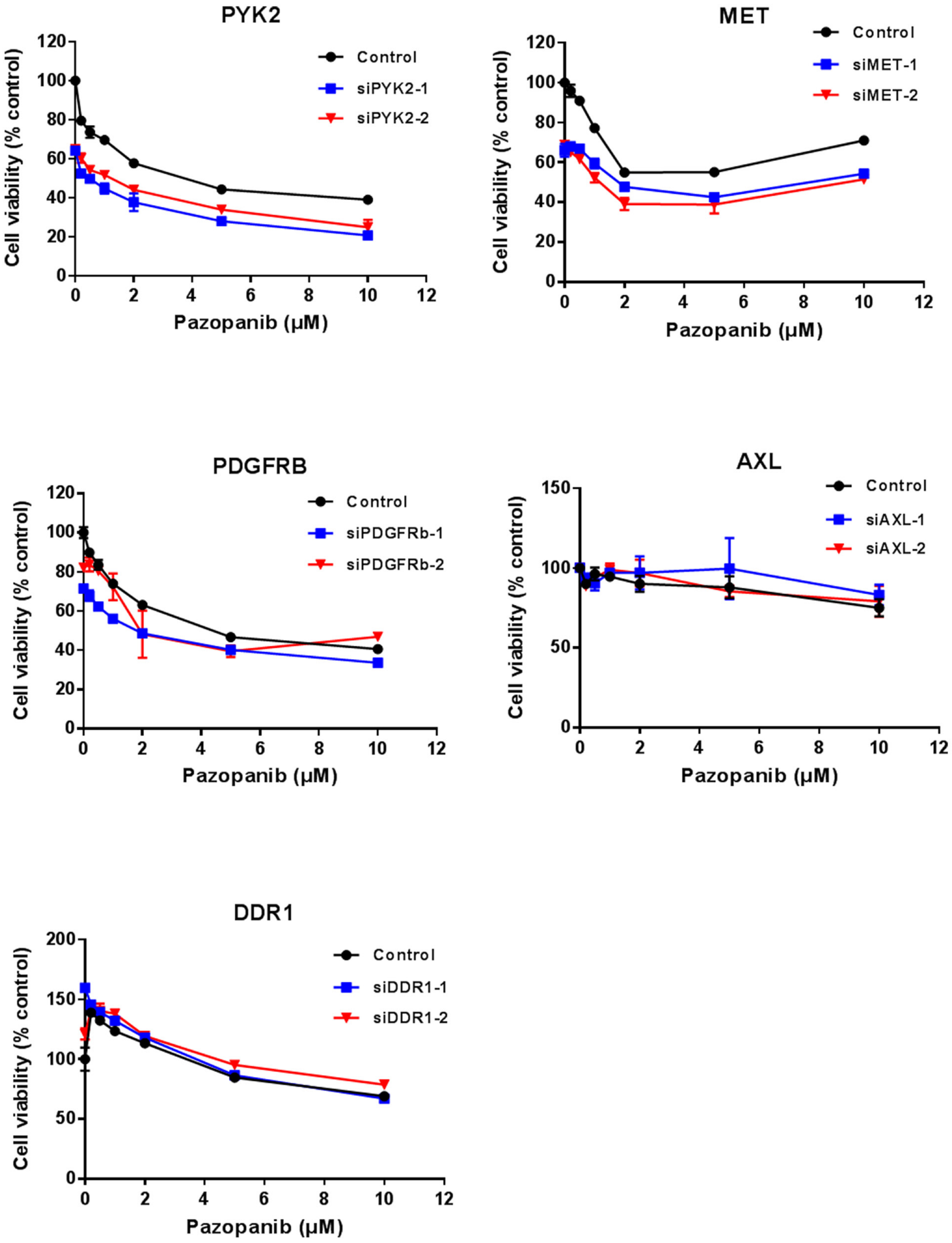

Supplementary Figure 4: Effect of a combination of siRNA-mediated gene silencing and pazopanibtreatment on cell proliferation of 1273/99 cell line.

Supplementary Table 1: List of antibodies used in western blotting.

See Supplementary File 1

Supplementary Table 2: List of siRNA sequences used in the study, used in experiments whose results are shown in Figure 3, Supplementary Figure 2, and Supplementary Figure 3.

See Supplementary File 2

Supplementary Table 3: Comparative analysis of pazopanib-sensitive and resistant cells using phosphotyrosine peptide substrate array.

See Supplementary File 3
